# Supplementary material for: Bayesian model selection for complex dynamic systems
Source: Nat Commun. 2018 May 4;9:1803. doi: 10.1038/s41467-018-04241-5 (PMC5935699; doi:10.1038/s41467-018-04241-5)
Supplement: Supplementary file 1 — Supplementary Information [file 41467_2018_4241_MOESM1_ESM.pdf]

Supplementary Information

Bayesian model selection for complex dynamic systems

Mark et al.

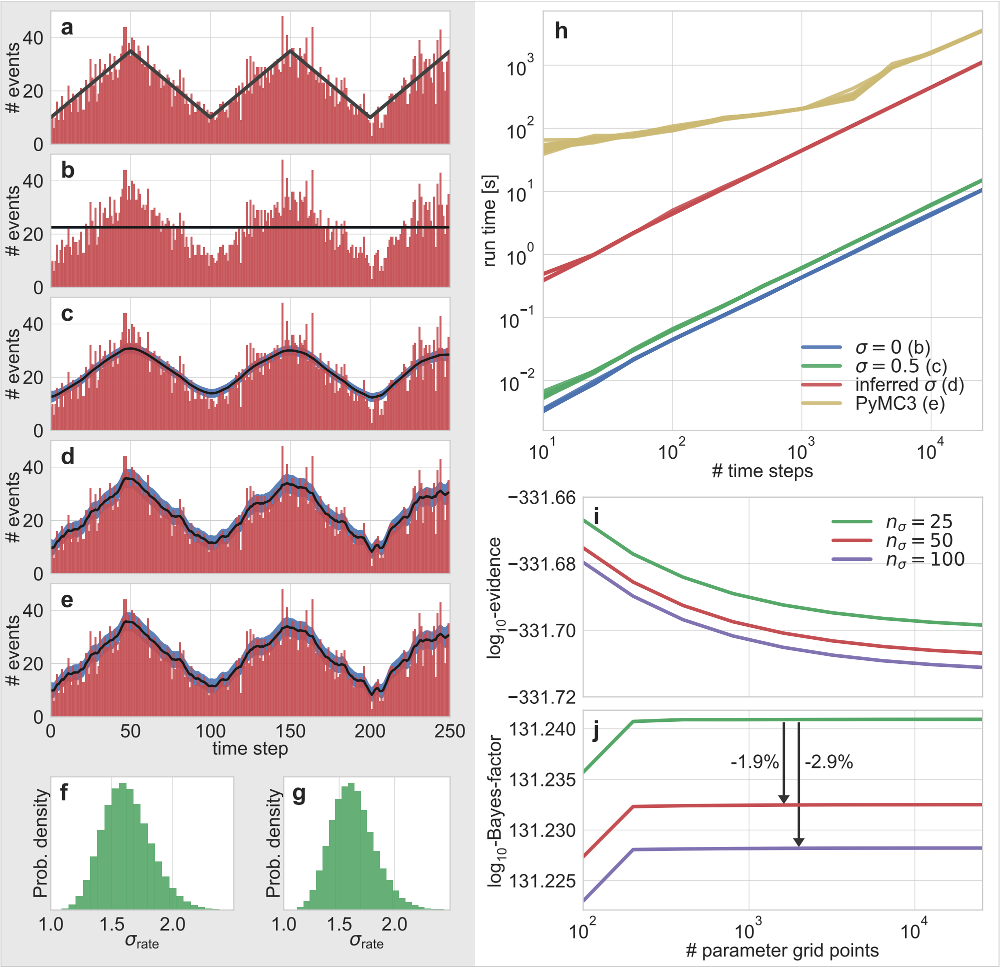

Supplementary Figure 1: **Performance evaluation of the grid-based inference approach.** **a:** Simulated Poisson process (red bars) with a linearly time-varying rate parameter λ (black line). For inference, we assume $\lambda \in]0, 60[$ in all cases, and discretize the rate parameter using 1000 equidistant values within this interval. **b:** Posterior mean value of inferred constant rate parameter (black line) and 90% credible interval (blue shading; barely visible). **c:** Posterior mean value of inferred time-varying rate parameter (black line) and 90% credible interval, assuming Gaussian parameter variations with fixed standard deviation $\sigma = 0.5$. **d:** Same as in (c), but with inferred high-level parameter σ. We discretize the high-level parameter σ using 50 equidistant values within the interval $]0, 3[$. **e:** Same as in (d), but fitted using the *No-U-Turn Sampler* [1], implemented in the *PyMC3* framework [2]. We draw 16500 samples from the posterior distribution and discard the first 1500 as "burn-in" samples to ensure proper tuning of the sampler. With this number of samples, the accuracy of the parameter estimates is comparable to the grid-based method. **f:** Inferred high-level parameter distribution $p(\sigma)$ corresponding to the grid-based inference in (d). **g:** Inferred high-level parameter distribution $p(\sigma)$ corresponding to the Monte-Carlo-based inference in (e). **h:** Run-time of the grid-based inference algorithm as a function of the number of simulated time steps (data points), assuming a static rate (blue), Gaussian rate fluctuations with a fixed (green) and inferred (red) standard deviation. The run-time of the Monte Carlo algorithm is depicted in yellow. **i:** Model evidence values corresponding to the analysis shown in (d), as a function of the number of grid points on the low-level parameter grid. Colors indicate a different number of grid-points on the high-level parameter grid. **j:** Bayes factors (the ratio of two model evidence values) comparing the time-varying parameter model (d) with the static parameter model (b), as a function of the number of grid-points on the low-level parameter grid. Colors indicate different numbers of grid-points on the high-level parameter grid (same as in (i)).

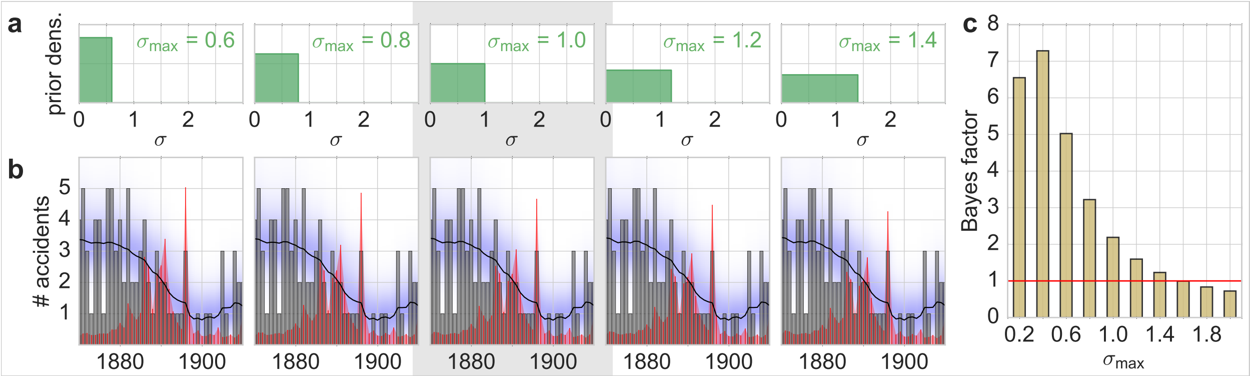

Supplementary Figure 2: **Flat high-level prior distributions in policy assessment. a:** To assess the influence of the chosen high-level prior distribution for the standard deviation σ of the gradual accident-rate fluctuations in the coal mining analysis, we analyze the data set using a flat prior distribution for σ with different maximum values σ_{max}. The analysis with $\sigma_{\text{max}} = 1$ is described in the main text and displayed in the center. **b:** As σ_{max} increases, the change-point distribution (red bars) is slightly smeared out, but the most probable year for the change-point remains unchanged. The choice of σ_{max} therefore does not influence the conclusions drawn from the change-point distribution. **c:** Model evidence values of the alternative model with varying σ_{max} divided by the model evidence values of the classic model, i.e. the Bayes factor in favor of the alternative model. Because a larger value σ_{max} generally results in a more flexible model and because the principle of Occam's razor penalizes overly flexible models, we observe a decreasing Bayes factor for large values of σ_{max}. However, some flexibility is needed to fit the data best. Therefore, constraining σ_{max} and hence the variations of the accident rate to overly small values also decreases the Bayes factor. We find that the alternative model is favored over the classic model within a wide interval of $0 < \sigma_{\text{max}} \lesssim 1.6$.

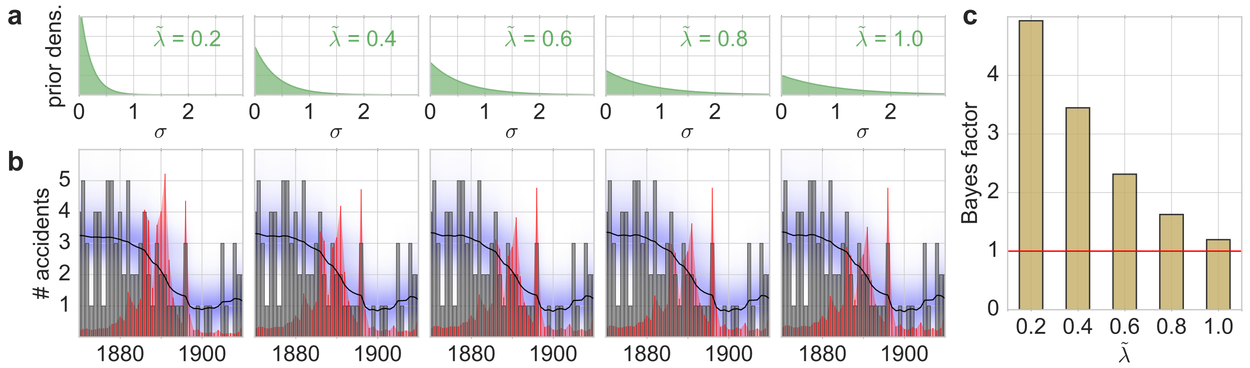

Supplementary Figure 3: **Exponential high-level prior distributions in policy assessment. a:** To test whether our conclusions from the coal mining analysis will change if we choose a different type of high-level prior distribution for the standard deviation of accident-rate fluctuations, we re-evaluate the analysis using exponential high-level priors with increasing rate parameter $\tilde{\lambda}$ (from left to right). **b:** We find that the most probable change-point remains in the year 1896 for $\tilde{\lambda} \gtrsim 0.4$. Only if we constrain the rate fluctuations tightly, as in the case $\tilde{\lambda} = 0.2$ (left), the earlier peak in the year 1891 becomes more probable compared to the peak in 1896. This is expected as the alternative model is bound to converge towards the classic model – which also favors the earlier peak in 1891 – as $\tilde{\lambda}$ gets smaller. **c:** Model evidence values of the alternative model divided by the model evidence values of the classic model, i.e. the Bayes factor in favor of the alternative model, for different values of $\tilde{\lambda}$. With increasing rate parameter $\tilde{\lambda}$, i.e. a wider high-level prior for σ, the Bayes factor in favor of the alternative model decreases. Up to an exponential high-level prior with $\tilde{\lambda} = 1.0$, the alternative model is favored compared to the classic model.

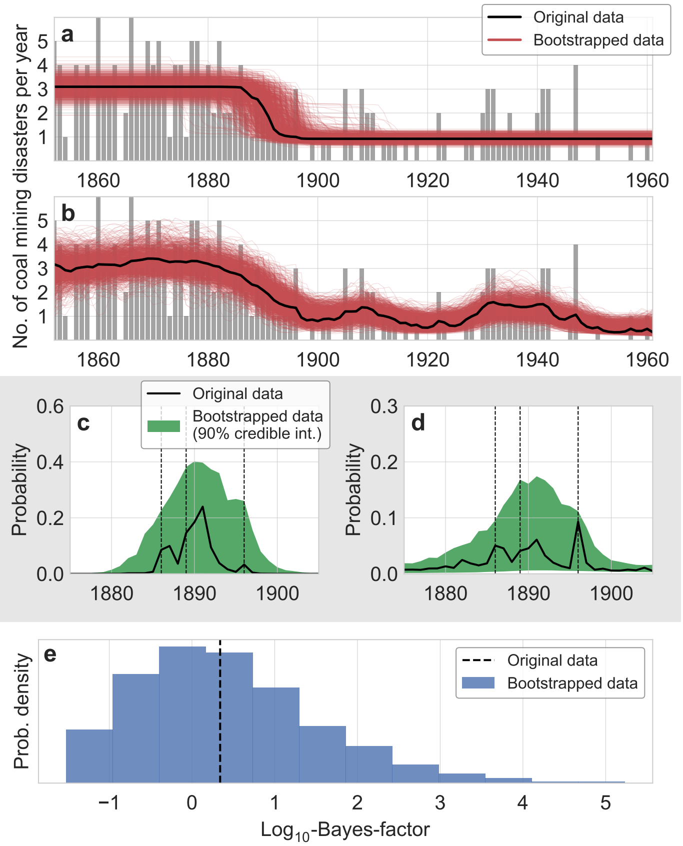

Supplementary Figure 4: **Verification of change-point models by bootstrapping.** To evaluate the accuracy of the inferred change-point distribution and the model evidence values of the coal mining example, we simulate 1250 datasets based on the inferred rate parameter values λ_t of the alternative change-point model. In particular, we draw a random number from a Poisson distribution with the time-varying rate parameter λ_t for each time step. The resulting time series is not identical to the original data, but it shares the same statistical properties. When we re-run the inference process on such a simulated dataset, the parameter estimates deviate from the original estimates. By repeating the analysis for many simulated datasets, we can thus quantify the variability of the estimates. **a:** Accident counts (gray bars) and the inferred accident rate (black line) for the actually observed dataset, based on the classic change-point model. The inferred accident rates of 1250 simulated datasets are denoted by red lines. **b:** Same as in (a), but for the alternative change-point model. **c:** Inferred change-point distribution based on the classic change-point model (black line), and the corresponding 90% credible interval based on the simulated datasets (green shading). The dashed lines correspond to the historically relevant events discussed in the main text (1886: publication of a report by the Royal Commission on Accidents in Mines, 1889: foundation of the Miner's Federation, 1896: enactment of the Explosives in Coal Mines Order and the Coal Mines Regulation Act). **d:** Same as in (c), but for the alternative change-point model. All inferred probability values of the change-point distribution lie within the 90%-credible interval of the bootstrapped change-point distributions, validating our interpretation of the individual peaks of the change-point distribution. **e:** Histogram of Bayes factors in favor of the alternative change-point model (on a $\log_{10}$-scale). The dashed line marks the Bayes factor obtained from the actually observed dataset. We find that the median Bayes factor of the simulated datasets (2.07) is close to the Bayes factor of the original dataset (2.18).

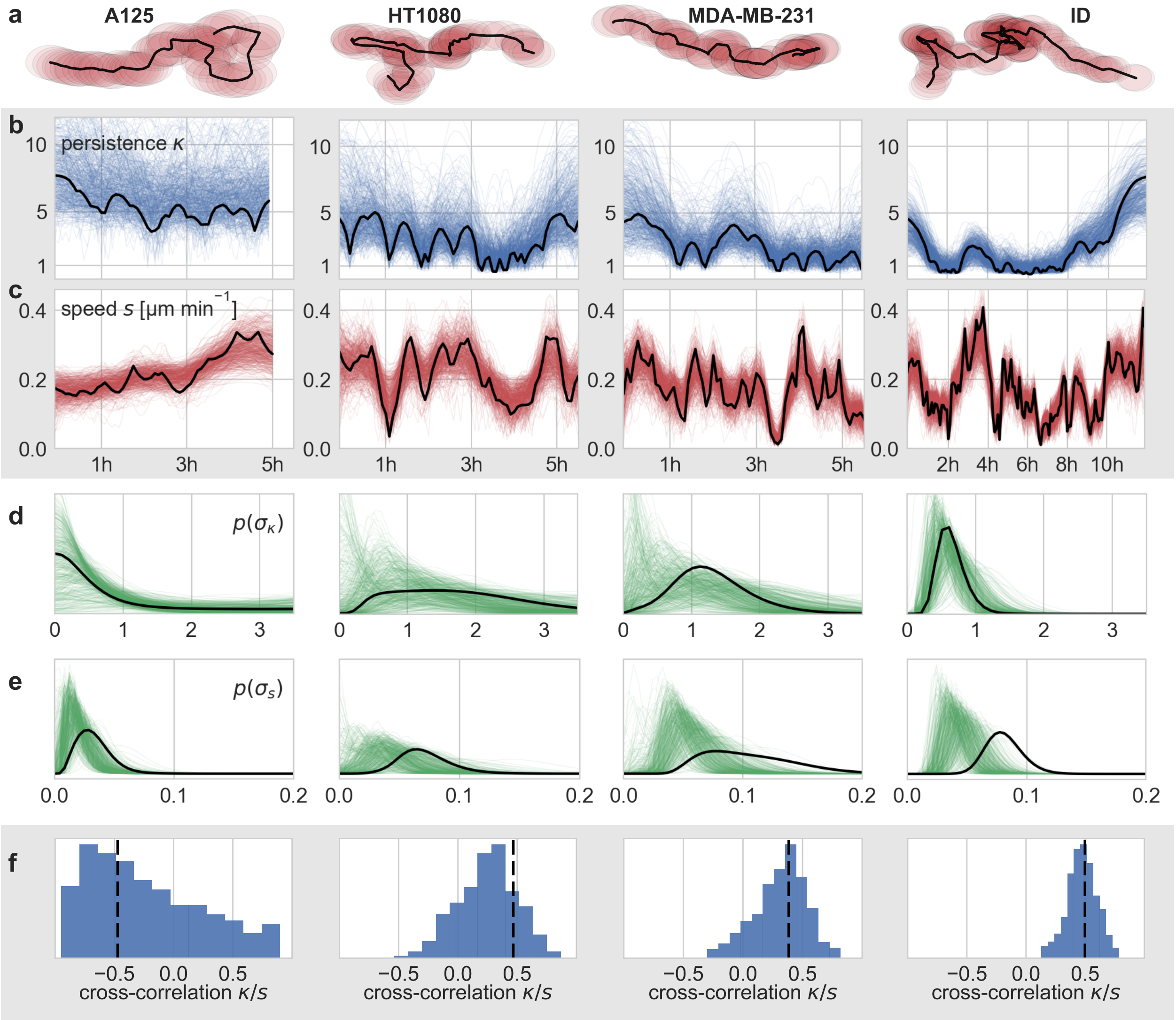

Supplementary Figure 5: **Verification of cancer cell random-walk analysis by bootstrapping.** To evaluate the accuracy of the inferred time-varying parameters and the cross-correlation coefficient between cell speed and directional persistence, we apply the bootstrapping technique to exemplary cell trajectories from all four cell types. In particular, we simulate 500 "hypothetical" trajectories for each actually observed trajectory, based on the inferred cell speed s_t and persistence κ_t. By repeating the analysis for many simulated datasets, we can thus quantify the variability of the parameter estimates. **a:** Exemplary cell trajectories for all four cell types. **b:** Inferred time-varying persistence κ_t for the observed trajectory (black) and 500 simulated trajectories (blue). **c:** Inferred time-varying cell speed s_t for the observed trajectory (black) and 500 simulated trajectories (red). **d:** Inferred magnitude of persistence fluctuations, σ_κ, for the observed trajectory (black) and 500 simulated trajectories (green). **e:** Inferred magnitude of speed fluctuations, σ_s, for the observed trajectory (black) and 500 simulated trajectories (green). **f:** Correlation coefficient of κ_t and s_t, for the observed trajectory (dashed line), and for 500 simulated trajectories (blue bars).

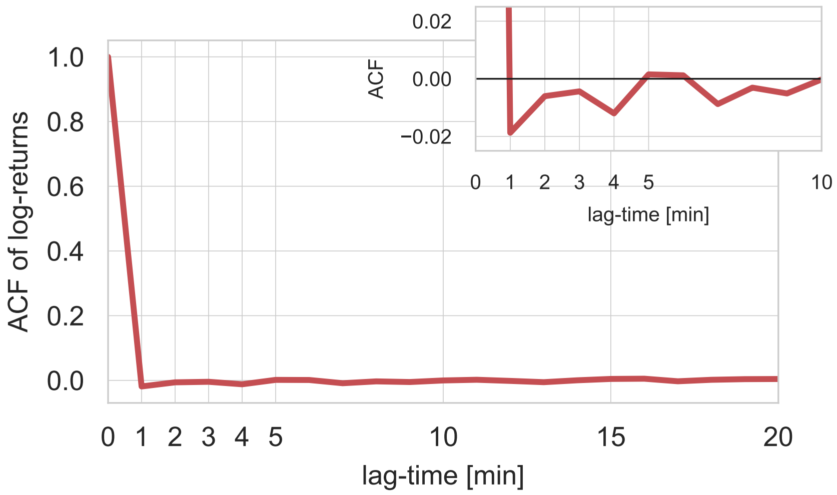

Supplementary Figure 6: Auto-correlation function of SPY log-returns, based on all trading minutes on regular trading days from 2011 to 2015. Inset: zoomed-in version to visualize weak negative correlation value for a lag-time of one minute.

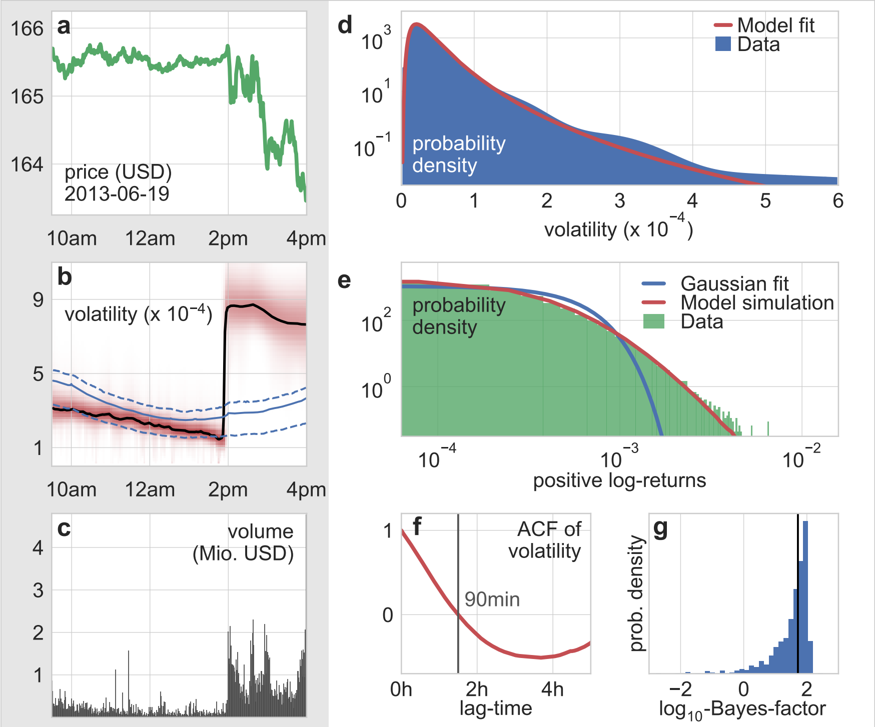

Supplementary Figure 7: **Volatility-only pricing model.** To evaluate the role of short-term correlations in the return series of SPY, we analyze the data based on a time-varying volatility model, but with zero short-term correlation $\rho = 0$. All other details of the model remain unchanged. **a:** Price evolution of SPY on June 19th, 2013. **b:** Inferred temporal evolution of the mean volatility (black line) and the corresponding parameter distribution (red shading). The blue line and dashed lines represent the average daily temporal evolution of the mean volatility and its inter-quartile range, respectively. **c:** Minute-scale trading volume of SPY. **d:** Inferred, time-averaged distribution of volatility (blue shading) and the corresponding fit using the same compound gamma distribution as in the main text (red line). **e:** Histogram of positive minute log-returns from the 2011 to 2015 of the exchange-traded fund SPY (green), together with the best fit Gaussian distribution of the data (blue line) and the simulated distribution of log-returns from the time-varying volatility model (red line). **f:** Auto-correlation function of the volatility parameter (red). **g:** Histogram of Bayes factors in favor of the volatility-only model. For each trading day, we compute the model evidence using the volatility-only model and divide this value by the corresponding model evidence value of the two-parameter model. On average, the volatility-only model is favored by a Bayes factor of 52, compared to the two-parameter model (black line). Note, however, that the model evidence does not consider long-range correlations between different parameters, as shown in Fig. 4h in the main text.

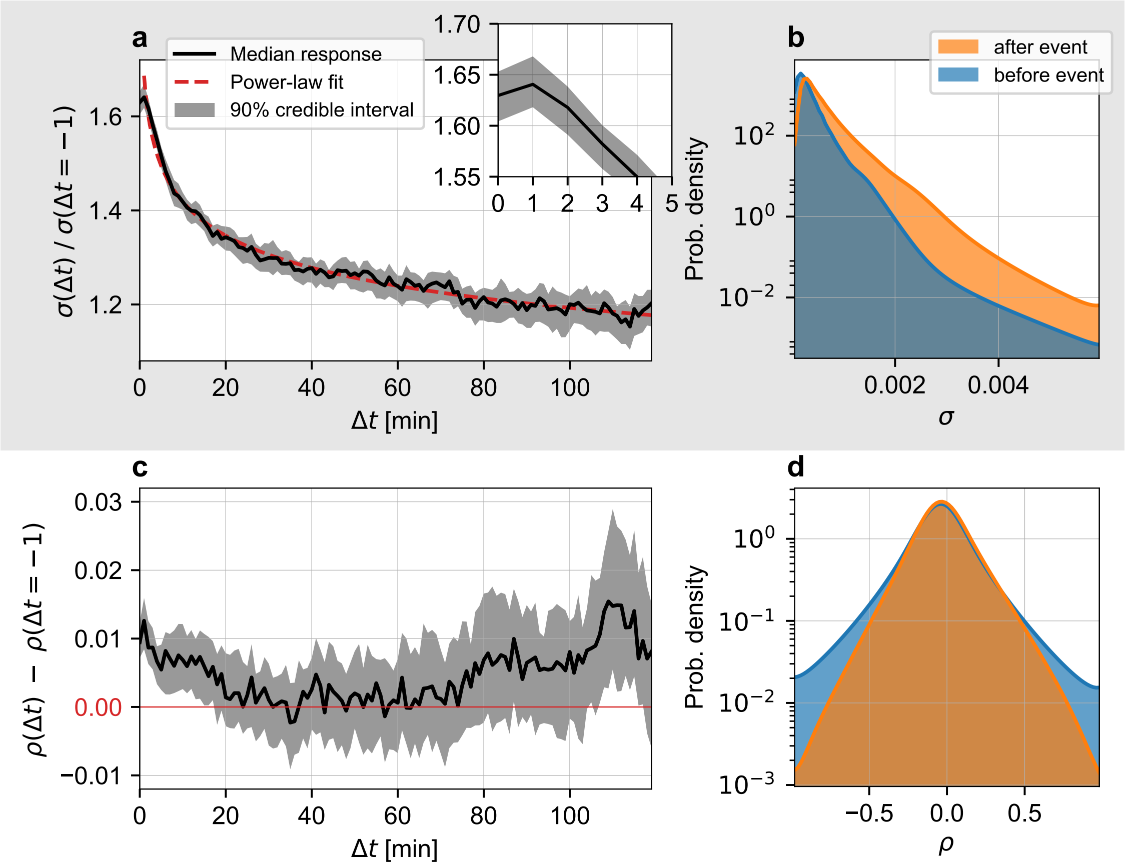

Supplementary Figure 8: **Response functions of time-varying correlation and volatility. a:** Temporal evolution of the stock market volatility σ_t after an event which contains a risk of at least 5% of rendering previous parameter estimates useless. We divide each series of estimated volatility values by the volatility value one minute before an irregular price change and subsequently compute the median based on all detected irregular price changes (black line). The error intervals depict the 90% credible interval of the median (gray shading). On average, the volatility is increased by a factor of 1.625 in the trading minute of the irregular pricing event and remains at that level for another minute (inset), before it decays over time with an estimated power-law coefficient of -0.076 (red dashed line). **b:** Averaged volatility parameter distribution from five trading minutes before an irregular pricing event, based on all detected events (blue). Averaged parameter distribution from the trading minute of an event and the following four minutes, based on all detected events (orange). As expected, larger volatility values are more probable after an event. **c:** Temporal evolution of the short-term correlation ρ after an irregular pricing event. From each series of estimated correlation values, we subtract the correlation value from one minute before the event and subsequently compute the median based on all detected events (black line). The error intervals depict the 90% credible interval of the median (gray shading). In contrast to the volatility, the median response function of the correlation shows no clear positive or negative deviation in reaction to an event. **d:** Same as in (b), but for the correlation parameter ρ. Interestingly, large absolute values of ρ are on average less likely after an event, compared to before an event.

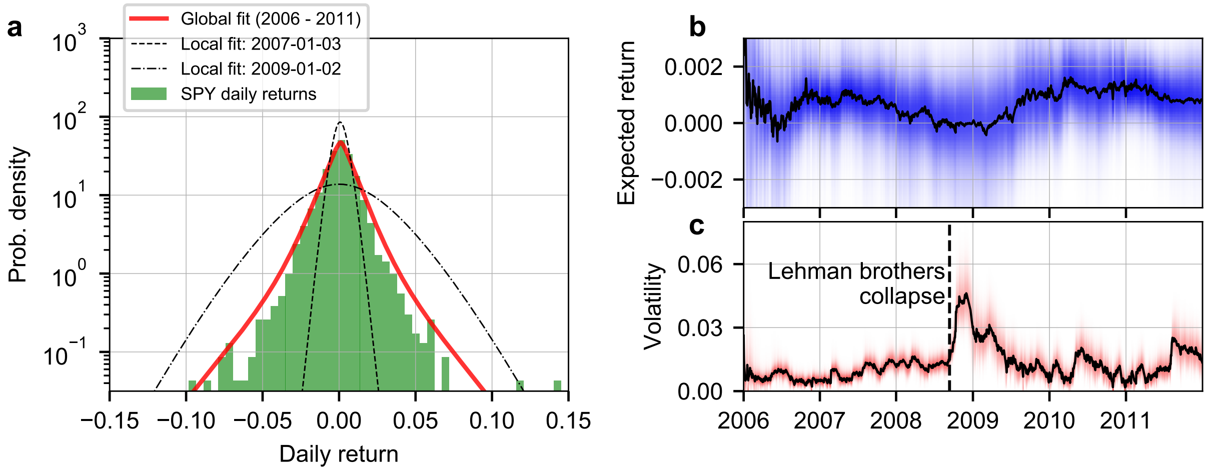

Supplementary Figure 9: **Time-varying distribution of returns.** The so-called "Value-at-Risk" (VaR) represents a widely employed indicator for risk. The VaR aims to estimate the maximum future loss (e.g. for the next day) of a financial asset that can only be exceeded with a small probability q. For example, if we choose $q = 5\%$, then the global VaR estimate is equal to the 5th percentile of the distribution of returns $p(r)$ (as only 5% of all returns will exceed the loss defined by the 5th percentile). Typical values for q are 1% and 5%. As the risk associated with a financial asset may change over time, there exist several ways of computing a local estimate of the VaR, including the sliding-window analysis and the return interval approach [3, 4, 5]. Here, we apply our online model selection approach discussed in the main text to estimate the local distribution of returns and thus the VaR. In particular, we describe daily returns r_t of the fund SPY by a Normal distribution, $r_t \sim N(\mu_t, \sigma_t)$, with a time-varying mean μ_t (the daily expected return) and time-varying standard deviation σ_t (the volatility). As in the example in the main text, we assume gradual variations of both parameters and furthermore expect one irregular pricing event per trading year. From the inferred values of μ_t and σ_t, we compute the local distribution of returns, which in turn allows us to evaluate the VaR. **a:** Histogram of all day-to-day returns of SPY from Jan 2006 to Dec 2011 (green), together with a global (red) and two local fits (black) based on the time-varying parameter model. **b:** Mean values (black line) and distributions (blue shading) of the time-varying expected return μ_t. **c:** Mean values (black line) and distributions (red shading) of the time-varying volatility σ_t.

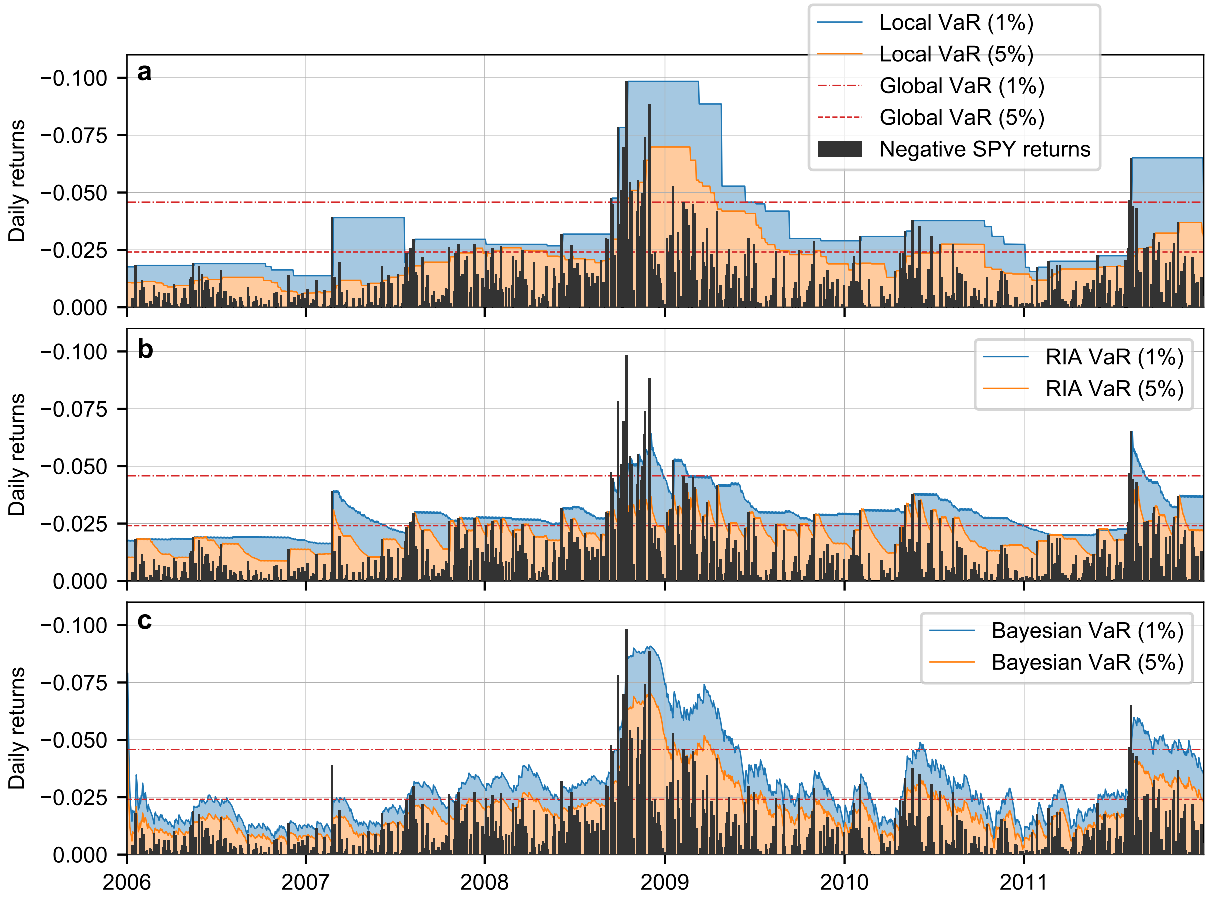

Supplementary Figure 10: **Dynamic Value-at-Risk estimation.** **a:** Classic Value-at-Risk (VaR) estimates for the SPY fund, with exceedance probability values of $q = 1\%$ and 5%. Global VaR estimates are based on all daily return values from Jan 2006 to Dec 2011. Local VaR estimates are based on the sliding window approach, i.e. the estimate for each day is based on the distribution of the last 100 return values. The local 1%-VaR estimates thus represent the maximum loss of the last 100 trading days. Note that the local 5%-VaR estimate substantially lags behind extreme events such as during the financial crisis of 2008/2009. **b:** Return-interval estimates of the local VaR, based on the algorithm described in [3, 4]. While these estimates in general increase promptly after major losses and only decrease slowly afterwards (as reported in [4]), they underestimate the risk tied to the financial crisis of 2008/2009 in the case of SPY. **c:** Bayesian estimates of the local VaR, based on the model described in Supplementary Fig. 9. Compared to the return-interval estimates, the Bayesian VaR shows a larger dynamic range, i.e. it attains larger risk values in times of major losses and smaller risk values in times of small losses. The Bayesian VaR further reacts promptly to big losses, but underestimates the VaR in some cases based on the prior assumption of only one irregular pricing event per year (c.f. Supplementary Fig. 9).

Supplementary References

[1] Hoffman, Matthew D and Gelman, A. The No-U-turn sampler: adaptively setting path lengths in Hamiltonian Monte Carlo. *J. Mach. Learn. Res.* **15**, 1593–1623 (2014).

[2] Salvatier, J., Wiecki, T. V. & Fonnesbeck, C. Probabilistic programming in Python using PyMC3. *PeerJ Comput. Sci.* **2**, e55 (2016).

[3] Bogachev, M. I. & Bunde, A. Improved risk estimation in multifractal records: Application to the value at risk in finance. *Phys. Rev. E* **80**, 026131 (2009).

[4] Ludescher, J., Tsallis, C. & Bunde, A. Universal behaviour of interoccurrence times between losses in financial markets: An analytical description. *Europhys. Lett.* **95**, 68002 (2011).

[5] Denys, M., Gubiec, T., Kutner, R., Jagielski, M. & Stanley, H. E. Universality of market superstatistics. *Phys. Rev. E* **94**, 042305 (2016).
