# Supplementary material for: Bayesian model selection for complex dynamic systems
Source: Nat Commun. 2018 May 4;9:1803. doi: 10.1038/s41467-018-04241-5 (PMC5935699; doi:10.1038/s41467-018-04241-5)
Supplement: Supplementary file 3 — Description of Additional Supplementary Files [file 41467_2018_4241_MOESM3_ESM.pdf]

Description of Additional Supplementary Files

File Name: Supplementary Data1
Description: Annual number of coal mining accidents in the United Kingdom involving ten or more men killed, from 1852 to 1961.

File Name: Supplementary Data 2
Description: Trajectories of tumor cells (A125, HT1080, MDA-MB-231, ID) migrating spontaneously on a fibronectin-coated substrate.
